# Global Overview of Youth Development: Comparison of the 5 Cs and Developmental Assets Across Six Countries

**DOI:** 10.3389/fpsyg.2021.685316

**Published:** 2021-07-23

**Authors:** Danielle Fernandes, Tina Pivec, Ayfer Dost-Gözkan, Fitim Uka, Margarida Gaspar de Matos, Nora Wiium

**Affiliations:** ^1^Department of Public Health and Primary Care, University of Ghent, Ghent, Belgium; ^2^Center for Evaluation Studies, Educational Research Institute Ljubljana, Ljubljana, Slovenia; ^3^Department of Psychology, Ozyegin University, Istanbul, Turkey; ^4^Department of Psychology, University of Prishtina, Prishtina, Kosovo; ^5^Center for Education and Health Promotion, University of Lisbon, Lisbon, Portugal; ^6^Department of Psychosocial Science, University of Bergen, Bergen, Norway

**Keywords:** positive youth development, adolescent, developmental assets, developmental psychology, cross-national, the 5Cs

## Abstract

Positive Youth Development (PYD) frameworks which describe young people's strengths and their relation to thriving and risk outcomes have gained significance among developmental researchers globally. As these models are being increasingly adopted, concerns remain about their generalizability outside of North America. It has been observed that the distribution and salience of assets differ for young people based on their cultural context. To better understand these varying developmental patterns, this paper studies the distribution of developmental assets and 5 Cs (Competence, Confidence, Character, Caring, and Connection) in youth from various countries and contrasting backgrounds. The total sample consisted of 4,175 students (62.5% females) with age ranging from 15 to 25 years (*M* = 18.95, *SD* = 2.49). 981 students were from Ghana (52.5% females), 900 students from Kosovo (66.7% females), 425 students from Norway (73.5% females), 247 students from Portugal (42.1% females), 648 students from Slovenia (63.4% females,), and 974 students from Turkey (68.7% females). Before comparisons of the countries, partial scalar invariance was confirmed. Analyses revealed that all countries differed in at least some internal or external developmental assets and at least in one of the 5 Cs. When considering internal assets, participants from Ghana seemed to have higher levels of internal assets together with participants from Norway who have the highest commitment to learning. Slovenian youth reported the highest levels of external assets of support and empowerment. Regarding the 5 Cs, Ghanaian youth reported having the highest confidence and character, and youth from Ghana, Kosovo, and Turkey are more caring and connected to others. The results uncovered unique patterns of PYD for each included country which are discussed through the lens of its political and social context. Through this focus on cross-national PYD patterns, this study advanced knowledge about the experiences of youth from a wide range of backgrounds and put forth suggestions for better policy measures and more culturally relevant interventions for optimal development of youth embedded in different cultures and countries.

## Introduction

The field of Positive Youth Development (PYD) has garnered increasing attention from developmental psychology researchers in the last two decades of the 20th century. During this time, PYD researchers challenged the notions of adolescence being a time of “storm and stress” and instead moved from this deficit view of development to focus on young people's strengths as promoters of optimal development (Damon and Gregory, 2003). This perspective of youth development has evolved from Bronfenbrenner's ecological systems theory (Bronfenbrenner and Mahoney, 1975) and developmental systems theories (Lerner et al., 2001). These theories focus on the alignment between individual development and the environmental context, with the bidirectional relations between the developing individual and his or her context regulating developmental outcomes.

Building on such contextual perspectives, Benson's Developmental Assets Framework (Benson, 2011) determines factors at the individual and environmental level which foster positive development. Forty such developmental assets have been identified, consisting of 20 internal assets and 20 external assets, which describe the values, relationships, resources, and skills young people need to achieve adequate development and effective functioning (Benson, 2011). Internal assets refer to an individual's skills and competencies, which include a commitment to learning, positive values, social competencies, positive identity (Benson, 2011). External assets represent positive features within an individual's environment, such as support, boundaries and expectations, empowerment, and constructive use of time (Benson, 2011). Search Institute researchers constructed this framework to provide a comprehensive view of development by outlining internal and external assets which can provide young people resilience and thus, prevent negative developmental outcomes such as poor mental health, low civic engagement, and unemployment (Scales and Taccogna, 2001). This framework of assets has been extensively used to assess the needs of young people and provides the necessary background to structure targeted interventions.

The Developmental Assets Profile (DAP) developed by the Search Institute to measure internal and external assets, has been used to survey ~3 million Grade 4 to Grade 12 American students over the past 3 decades and demonstrate that these assets were closely linked with higher levels of thriving (Benson et al., 2011). Studying the distribution of developmental assets has acted as a foundation for developing intervention programs that use an asset-building strategy. One such intervention, the “Building Assets Reducing Risk” (BARR) program supported by the US Department of Education, promoted developmental assets among students which led to improved academic performance, students' experiences, and teachers' satisfaction (Bos et al., 2019). The Search Institute's flagship program, “Asset-Getting to Outcomes” (AGTO), also successfully incorporated the developmental assets framework in the existing Getting to Outcomes program (a 10-step youth implementation model) to build the community's capacities to implement youth programs and achieved enhanced developmental outcomes (Chinman et al., 2012).

Another pivotal theoretical concept within the PYD framework is the 5 Cs model, which represents a multi-faceted perspective of positive development. This developmental systems theory draws from extensive reviews of youth development literature, to operationalize the concept of “thriving” through an assessment of 5 “Cs”: Competence, Confidence, Character, Connection, and Caring (Lerner et al., 2005). Competence describes a positive view of one's abilities and actions; Confidence represents an individual's internal sense of positive self-worth and self-efficacy; Connection refers to positive bonds with family, friends, and the broader community; Character indicates an individual's sense of respect for morals and values, and Caring describes a person's sense of sympathy and empathy for others (Lerner et al., 2005). The model postulates that these lower-order factors contribute to overall development in a linear fashion. An extensive longitudinal study, the 4-H Study of PYD (Lerner et al., 2011), provided empirical support for this model among American youth. Global PYD research has also demonstrated the validity of this model to predict positive developmental outcomes, as well as protect against risk and problem behaviors (Bowers et al., 2010; Lerner et al., 2013).

The tenets of the 5 Cs model, particularly its emphasis on the mutually influential and bidirectional relationship between an individual and their context, have been used to frame youth development programs in North America (Harwood et al., 2015; Brandes, 2017). One such program, the Green Care Intervention is a nature-based program that uses animal-assisted and horticulture interventions as a framework to promote positive developmental outcomes (Brandes, 2017). This program was piloted among 20 students at a special education school in New York, and through qualitative investigation, the investigators found that the youth experienced a change in the 5 Cs domains after exposure to the animal- intervention. Another intervention developed for young soccer players involved coaches using strategies relevant to the 5 Cs in their coaching sessions at the youth sports academy (Harwood et al., 2015). Self-reported data from the players corroborated by observational assessments by the coaches and parents indicated psychosocial improvements.

As the PYD frameworks grew in popularity in North America, research indicated that while developmental assets were observed across cultures, their significance for specific developmental outcomes varied among different ethnic and racial groups (Scales et al., 2000; Sesma et al., 2003; Holsen et al., 2017). An example of this can be seen through the Search Institute's cross-sectional survey with a diverse group of African American, American Indian, Asian American, Latino/Latina, White, and Multiracial youth, which determined that there are both similarities and key differences in how developmental assets are interpreted and applied among these different groups (Sesma et al., 2003). Across the sample of 217,277 6th- to 12th-grade students, the presence of a higher number of assets was associated with lower engagement in high-risk behavior and a higher likelihood of thriving outcomes. Thus, the developmental assets played an important role in both prevention and promotion, irrespective of racial or ethnic backgrounds. Certain assets also demonstrated a consistent influence on developmental outcomes across ethnic groups. For example, internal assets such as achievement motivation and school engagement in the “commitment to learning” domain had a significant relationship with academic outcomes, and support was associated with improved health and well-being among all youth. However, the survey findings also indicated that assets may have a stronger relationship with specific developmental outcomes based on certain racial/ethnic differences. While the “constructive use of time” asset was strongly related to school success for American Indian and Asian American youth, a significant association was not observed in other racial/ethnic groups. Similarly, support played a more powerful role in preventing anti-social behavior among American Indian and White youth, as compared to their peers. These culture-specific differences in the relationship between developmental assets and outcomes were further described in a study with 6,000 students (Scales et al., 2000), representing six different ethnic groups. This study determined that self-esteem and reading for pleasure were more important for African American youth to achieve overall thriving while caring and engaging in creative activities were significant for American Indian youth.

Differences between ethnic groups in the US are only one aspect of the diversity to be expected when operationalising PYD frameworks in young people's lives. As these frameworks are being increasingly adopted globally, developmental researchers need to explore these contextual differences and use this background to inform asset-building initiatives. The first forays into global PYD research have largely been efforts to understand the differences in asset distribution between youth from different parts of the world. One such cross-national study (Scales, 2011) among young people in Albania, Bangladesh, Japan, Lebanon, and the Philippines showed that young people in Bangladesh, the Philippines, and Japan reported lower scores on most internal and external assets, compared to their counterparts. Albanian youth had higher levels of commitment to learning, while positive values and social competencies were predominant in Lebanon.

The Search Institute's pioneering work in adapting the developmental assets framework in more than 30 countries, has also uncovered evidence on the relationships between these assets and developmental outcomes (Scales et al., 2017). Their findings indicate that young people around the world do better when they have higher levels of developmental assets, with thriving outcomes encompassing mental and physical health, academic success, and civic engagement even in high-risk or challenging environments. Family and school-related assets proved to be significant contributors to well-being in global youth, while communities and neighborhoods were often described as least conducive to providing young people with the assets they need to thrive. Other studies validating the 5 C model in different countries have also indicated differences in asset distribution and the way they influence developmental outcomes. Studies from Norway reported that higher scores on caring had a positive correlation with anxiety and depressive symptoms (Holsen et al., 2017). Cross-national studies between Norway and Turkey found that while Norwegian youth had high social competency, Turkish youth scored high for positive identity (Wiium et al., 2019). Studies from Ghana demonstrated that young people have a higher distribution of internal assets and a comparative lack of external assets (Wiium, 2017).

Understanding these cultural differences and accommodating them in the intervention-development process is essential to effectively promote the healthy development of children and adolescents. An excellent example of this is “Project Venture,” a program that moved past the deficit-focussed approach to American Indian/Alaska Native (AI/AN) youth, to incorporate cultural practices and reinforce traditional values through activities, like storytelling, to build PYD assets (Kenyon and Hanson, 2012). This approach showed significant effects on reducing substance abuse among these youth. The success of this intervention speaks to the potential of operationalizing strengths or assets that are dominant in a specific community to structure a program.

Awareness of how an individual's cultural context may influence their participation is another essential aspect of developing contextually appropriate interventions. A notable example of this is the efforts initiated by the “Assets for Colorado Youth” organization, to build assets in communities of color through culturally-specific approaches, such as translating and re-interpreting the assets framework into different languages and relating each asset to traditional Mexican proverbs and quotes (Lucero, 2000). The development of the PLAAY project and the “Building Community Strengths” project, both focussing on racial and ethnic minorities in the US, demonstrated the importance of considering young people's socio-cultural backgrounds (Stevenson, 2003; Letiecq and Bailey, 2004). Researchers with the PLAAY project were required to remain mindful of Black teen's day-to-day encounters with racial discrimination and community violence, and its influence on their engagement with the intervention (Stevenson, 2003). The “Building Community Strengths” project acknowledged the historically negative treatment of American Indian communities by researchers and adopted a participatory approach through close engagement with local community leaders (Letiecq and Bailey, 2004).

While there is emerging global research on the validity of the development assets and 5 Cs model and their associations with developmental outcomes among youth from different nationalities, there is a dearth of literature exploring the relevance of developmental assets in the development of contextually-appropriate interventions. This study aims to provide a global perspective on the distribution of developmental assets and the 5 Cs in youth from vastly different socio-cultural contexts and the significance of these assets for intervention development. This study provides a contextual focus while examining the differences in developmental strengths (i.e., developmental assets and the 5 Cs) among young people from six countries; Ghana, Kosovo, Norway, Portugal, Slovenia, and Turkey, which represent diverse economic and socio-political contexts. The distributions of strengths are discussed in the light of each country's youth-focused initiatives, cultural background, and social climate. Through a study of developmental assets and the 5 Cs among young people from different countries, this paper will lay a foundation for the development of youth-focused programs that are both culturally-relevant and grounded in the PYD framework.

## Methods

### Participants

The total sample of the present study consisted of participants from seven countries (*N* = 4,175 (62.5 % females) with age ranging from 15 to 25 years (*M* = 18.95, *SD* = 2.49). The sample included 981 students from Ghana (52.5% females, *M*_*age*_ = 19.82, *SD* = 1.74), 900 students from Kosovo (66.7% females, *M*_*age*_ = 16.34, *SD* = 0.97), 425 students from Norway (73.5% females, *M*_*age*_ = 20.16, *SD* = 1.51), 247 students from Portugal (42.1% females, *M*_*age*_ = 16.60, *SD* = 1.29), 648 students from Slovenia (63.4% females, *M*_*age*_ = 19.81, *SD* = 2.63), and 974 students from Turkey (68.7% females, *M*_*age*_= 19.96, *SD* = 2.46).

### Instruments

#### Developmental Assets

Developmental assets were measured using The Developmental Assets Profile (DAP) tool (Benson, 2003), which consists of items assessing young people's experience of developmental assets. They are divided into internal and external assets categories. The external assets include Support (seven items, e.g., “I have a family that gives me love and support”), Empowerment (six items, e.g., “I feel valued and appreciated by others.”), Boundaries and expectations (nine items, e.g., “I have friends who set good examples for me.”), and Constructive Use of Time (four items, e.g., “I am involved in creative things such as music, theater or other arts.”). The internal assets consist of Commitment to Learning (seven items, e.g., “I enjoy learning.”), Positive Values (seven items, e.g., “I tell other people what I believe in.”), Social Competencies (seven items, e.g., “I accept people who are different from me.”), and Positive Identity (eight items, e.g., “I am sensitive to the needs and feelings of others.”). Responses were rated on a 4-point Likert scale (1 = not at all or rarely, 4 = extremely or almost always). Reliability measures (Cronbach's alphas) of the developmental assets were adequate except for the subscale Constructive use of time: Support (Ghana:0.72; Kosovo:0.75; Norway:0.80; Slovenia:0.74; Turkey:0.81); Empowerment (Ghana:0.76; Kosovo:0.60; Norway:0.76; Slovenia:0.72; Turkey:0.73); Boundaries and expectations (Ghana:0.81; Kosovo:0.77; Norway:0.78; Slovenia:0.71; Turkey:0.80); Constructive use of time (Ghana:0.51; Kosovo:0.50; Norway:0.40; Slovenia:0.41; Turkey:0.52); Commitment to learning (Ghana:0.83; Kosovo:0.80; Norway:0.84; Slovenia:0.72; Turkey:0.84); Positive values (Ghana:0.80; Kosovo:0.69; Norway:0.67; Slovenia:0.60; Turkey:0.67); Social competences (Ghana:0.81; Kosovo:0.73; Norway:0.76; Slovenia:0.72; Turkey:0.76); Positive identity (Ghana:0.76; Kosovo:0.74; Norway:0.87; Slovenia:0.83; Turkey:0.85). The reported reliabilities are consistent with previous studies (Scales, 2011).

#### The 5Cs

The short form of the PYD questionnaire (Geldhof et al., 2014) was used to measure the 5Cs (i.e., Competence, Confidence, Character, Caring, and Connection). It consists of 34 items answered on a 5-point Likert scale (with responses ranging from 1 = strongly disagree to 5 = strongly agree). Sample items that measure the 5Cs are: Competence (e.g., I do very well in my classwork at school); Confidence (e.g., All in all, I am glad I am me); Character (e.g., I hardly ever do things I know I shouldn't do); Connection (e.g., My friends care about me); and Caring (e.g., When I see another person who is hurt or upset, I feel sorry for them). Reliability measures (Cronbach's alphas) of the 5Cs are adequate: Competence (Ghana:0.70; Kosovo:0.67; Portugal:0.80; Slovenia:0.67; Turkey:0.74); Confidence (Ghana:0.88; Kosovo:0.76; Portugal:0.87; Slovenia:0.89; Turkey:0.89); Character (Ghana:0.80; Kosovo:0.68; Portugal:0.72; Slovenia:0.65; Turkey:0.71); Caring (Ghana:0.86; Kosovo:0.84; Portugal:0.87; Slovenia:0.82; Turkey:0.87); Connection (Ghana:0.83; Kosovo:0.74; Portugal:0.83; Slovenia:0.76; Turkey:0.80). The reported reliabilities are consistent with previous studies (Geldhof et al., 2014).

### Procedure

The data was gathered in six different countries (Ghana, Kosovo, Norway, Portugal, Slovenia, and Turkey), however, only participants from Ghana, Kosovo, and Slovenia completed both Developmental assets profile and the 5 Cs of Positive Youth Development Questionnaire. Half of the participants from Turkey completed the Developmental assets profile and the other half completed the Positive Youth Development Questionnaire. Participants from Norway completed only the Developmental Assets Profile and participants from Portugal completed only the PYD questionnaire.

In Ghana, cross-sectional data were collected from 1st-year students at three state universities: University of Development Studies, Kwame Nkrumah University of Science and Technology, and the University of Ghana. Participants were selected through convenience sampling in three different regions in Ghana: the Northern, the Ashanti (in mid-Ghana), and the Greater Accra region in the South, respectively. A paper-and-pencil self-administered questionnaire was completed by participants, who received a pen as a small token. Informed consent was obtained from students before their voluntary participation in the study. The study was approved by the Ethics Committee for Humanities at the University of Ghana.

In Kosovo, school principals, teaching staff, parents, and students were informed about the purpose and methods of the study prior to data collection. Upon agreement by schools to take part in the study, parental and student consent was obtained. After that, every participant completed the study measures as an anonymous self-report questionnaire at their schools, during their regular school hours in person. Two well-trained psychologists administered data collection and informed/supported students when the questionnaire was being filled out in a group setting. The procedure of data collection per class took ~45 min. We obtained the IRB from the University of Prishtina. Participants were not compensated for their time.

In Norway, cross-national data were collected from a convenience sample of 1st-year students at the Faculties of Mathematics and Natural Sciences, Psychology, Law, Social Sciences, and Medicine at the University of Bergen. A specialized company in interpretation services (Semantix Translations Norway AS), translated the original English questionnaire to Norwegian. A web-based self-administered questionnaire was completed by participants. Informed consent was obtained from students before their voluntary participation in the study. The study was approved by the Regional Committee for Medical and Health Research Ethics (REK) in Norway.

In Portugal, a questionnaire was approved by the Santa Maria Hospital Ethics Committee. Before the data collection, informed consent was obtained from parents or caregivers in the case of adolescents under the age of 18 years. Participants were not compensated for their time.

In Slovenia, data were collected from a convenience sample of high school and university students. Before the data collection, parents and students were informed about the goal and purpose of the study and informed consent was acquired from parents or participants (depending on the age of the students). After that, half of the participants completed the study as an anonymous self-report questionnaire in their schools in a paper-pencil form or an online survey. Participants were not compensated for their time. The study was approved by the local ethical research committee.

In Turkey, data were collected using Qualtrics. Before the data collection, participants were informed about the goal and procedures of the study. At the beginning of the questionnaire, informed consent was obtained from the participants. The study was approved by The Ethical Board of Ozyegin University. The survey was anonymous and participants could withdraw from participation in the survey without any penalty. Students were given extra credit for their participation in research.

### Statistical Analysis

For each country separately, descriptive analyses were run on the demographic variables, skewness, and kurtosis were checked, and the reliability tests were done for each asset category and each PYD outcome. Furthermore, mean scores were used for developmental assets and the 5Cs. Before comparing the countries across PYD outcomes, Multigroup Confirmatory Factor Analysis (MGCFA) was applied in the Mplus program, version 8.6 (Muthén, L. K., and Muthén, 1998/2021). We used ESEM (Exploratory Structural Equation Modeling) instead of CFA (Confirmatory Factor Analysis) as it allows the pre-specification of target and non-target loadings, while all non-target loadings are close to 0 and are not fixed as 0 as in the case in the CFA (Morin et al., 2016). The ESEM approach is similar to CFA, however, it is less constraining. Model fit was assessed with chi-squares, Comparative Fit Indices (CFI), Root-Mean-Square Error of Approximation (RMSEA), and Standardized Root-mean-square Residual (SRMR), following a recommendation from Hu and Bentler (Hu and Bentler, 1999) for a good fit: CFI >0.95, RMSEA <0.06, and the SRMR <0.08. For adequate fit the following cut off values were applied: CFI >0.90, RMSEA <0.08, and the SRMR <0.08 (Hair et al., 1998). Full information maximum likelihood (FIML) algorithm was used to handle missing data and assess parameters in the model. Lastly, to compare the differences across countries in the developmental assets and the 5Cs, MANCOVA was employed. Before the analyses, outliers were deleted from the dataset. After meeting the recommendations for the MANCOVA, two MANCOVA analyses with Bonferroni correction were carried out. Thus, the country was treated as an independent variable, the developmental assets, and the 5Cs as the dependent variables, while gender and age were the control variables.

## Results

### Descriptive Statistics

Descriptive statistics (*M, SD*) for developmental assets together with correlations for the whole sample are presented in Table 1. All internal and external developmental assets are positively correlated with values from 0.17 to 0.66.

**Table 1:** Descriptive statistics and correlations among the developmental assets categories for the whole sample (*N* = 4,010).

	***M***	***SD***	**1**	**2**	**3**	**4**	**5**	**6**	**7**
1. Support	2.94	0.56							
2. Empowerment	3.19	0.53	0.57^***^						
3. Boundaries and expectations	3.00	0.52	0.66^***^	0.56^***^					
4. Constructive use of time	2.28	0.67	0.25^***^	0.33^***^	0.27^***^				
5. Commitment to learning	3.25	0.55	0.39^***^	0.42^***^	0.48^***^	0.23^***^			
6. Positive values	3.43	0.45	0.33^***^	0.34^***^	0.40^***^	0.17^***^	0.47^***^		
7. Social competences	3.20	0.50	0.37^***^	0.44^***^	0.45^***^	0.29^***^	0.56^***^	0.57^***^	
8. Positive identity	3.16	0.69	0.37^***^	0.38^***^	0.40^***^	0.27^***^	0.41^***^	0.42^***^	0.50^***^

* *p < 0.05,*

** *p < 0.01,*

*** *p < 0.001*.

In Table 2, descriptive statistics (*M, SD*) for 5 Cs together with correlations for the whole sample are presented. All 5 Cs are positively correlated with values from 0.23 to 0.54.

**Table 2:** Descriptive statistics and correlations among the 5 Cs for the whole sample (*N* = 3,790).

	***M***	***SD***	**1**	**2**	**3**	**4**
1. Competence	3.51	0.69				
2. Confidence	3.95	0.78	0.54^***^			
3. Character	4.03	0.59	0.33^***^	0.42^***^		
4. Caring	4.19	0.76	0.23^***^	0.24^***^	0.53^***^	
5. Connection	3.79	0.66	0.47^***^	0.48^***^	0.45^***^	0.37^***^

* *p < 0.05,*

** *p < 0.01,*

*** *p < 0.001*.

### Multi-Group Confirmatory Factory Analysis

In Table 3, fit indexes of MGFCA that was used to ascertain measurement invariance for developmental assets are presented. The configural invariance model showed adequate fit, demonstrating that similar patterns of eight categories of developmental assets and latent constructs were observed across Ghana, Kosovo, Norway, Slovenia, and Turkey. In the metric invariance model, where factor loadings were constrained to be equal across all countries, fit indices showed adequate fit. In the scalar invariance model, where variables were constrained to have equal intercepts across countries, an adequate model was not achieved. Following the modification indices, means of empowerment, boundaries and expectations, commitment to learning, and social competencies were allowed to vary across countries, therefore, partial scalar invariance was attained.

**Table 3:** Measurement invariance models and goodness-of-fit indexes of multigroup analyses of developmental assets across countries.

**Model**	**Model fit indices**			
	**χ^**2**^ (df)**	**RMSEA**	**90% CI RMSEA**	**CFI**
Configural invariance	10899.20 (5,283)	0.037	0.036–0.038	0.916
Metric invariance	12604.13 (5,912)	0.038	0.037–0.039	0.900
Scalar invariance	12889.58 (6,084)	0.052	0.051–0.053	0.809
Partial scalar invariance	12702.48 (5,970)	0.038	0.037–0.039	0.900

*χ^2^, Chi-square; df, degrees of freedom; CFI, Comparative Fit Index; RMSEA, root mean square error of approximation; CI, confidence interval*.

In Table 4, fit indexes of MGCFA of the 5Cs are presented. The configural invariance and metric invariance model indicated adequate fit. In the scalar invariance model, we failed to achieve an adequate or good fit. After following the modification indices, means for confidence, character, and connection were allowed to vary across countries, after that, partial scalar invariance was achieved.

**Table 4:** Measurement invariance models and goodness-of-fit indexes of multigroup analyses of the 5Cs across countries.

**Model**	**Model fit indices**			
	**χ^**2**^ (df)**	**RMSEA**	**90% CI RMSEA**	**CFI**
Configural invariance	5269.06 (1,991)	0.055	0.053–0.057	0.920
Metric invariance	6145.50 (2,547)	0.051	0.049–0.052	0.905
Scalar invariance	8519.14 (2,663)	0.063	0.062–0.065	0.845
Partial scalar invariance	6306.21 (2,625)	0.051	0.049–0.052	0.903

*χ^2^, Chi-square; df, degrees of freedom; CFI, Comparative Fit Index; RMSEA, root mean square error of approximation; CI, confidence interval*.

### Comparison Across Countries

To examine the differences in developmental assets across countries, MANCOVA was employed (Λ = 0.60; *F* = 65.92; *p* < 0.001; partial η^2^ = 0.12). In Table 5, the means of the developmental assets are presented together with standard errors. The Bonferroni *post-hoc* tests revealed that included countries significantly differed in all examined developmental assets. Regarding the external developmental assets, participants from Slovenia reported higher Support than participants from Ghana (*p* = 0.005) and participants from Kosovo (*p* < 0.001). There were no differences among other countries. Moreover, participants from Slovenia reported the highest levels of Empowerment as well since they differed from all included countries (*p*s < 0.001) and were followed by Norway and Turkey which were no different from one another (*p* = 0.582). Participants from Ghana reported higher Empowerment than participants from Kosovo (*p* = 0.016). The latter had the lowest Empowerment among all countries. For Boundaries and expectations, the picture is not that clear. Participants from Ghana had the highest level of Boundaries and expectations, however, participants from Ghana and Norway did not differ from one another (*p* = 1.00). Participants from Norway were no different from participants from Slovenia. Participants from Kosovo and Turkey had the lowest levels of Boundaries and expectations. Comparison of countries in Constructive use of time revealed that participants from Ghana, Slovenia, and Turkey (*p* > 0.05) used their time most constructively, followed by participants from Norway, although participants from Turkey and Norway did not differ from one another. Participants from Kosovo reported they used their time least constructively.

**Table 5:** Developmental assets by Country: MANCOVA.

**Variable**	**Ghana^**a**^*M (SE)***	**Kosovo^**b**^*M (SE)***	**Norway^**c**^*M (SE)***	**Slovenia^**d**^*M (SE)***	**Turkey^**e**^*M (SE)***	***SS***	***df***	***MS***	***F***	**Country differences**
Support	2.92 (0.02)	2.89 (0.02)	2.96 (0.03)	3.03 (0.02)	2.95 (0.02)	6.12	4	1.53	4.98^**^	d > b, c
Empowerment	3.11 (0.02)	3.02 (0.02)	3.29 (0.03)	3.47 (0.02)	3.23 (0.02)	76.28	4	19.07	74.32^**^	d > c, e > a > b
Boundaries and expectations	3.09 (0.02)	2.97 (0.02)	3.09 (0.03)	3.00 (0.02)	2.92 (0.02)	18.10	4	4.52	17.64^**^	a, c (= d) (= b) (= e)
Constructive use of time	2.46 (0.02)	1.87 (0.03)	2.28 (0.03)	2.42 (0.03)	2.38 (0.02)	131.42	4	32.86	80.55^**^	a, d, e > c (= e) > b
Commitment to learning	3.35 (0.02)	3.14 (0.02)	3.45 (0.03)	3.11 (0.02)	3.27 (0.02)	42.58	4	10.65	37.90^**^	c > a > e > b, d
Positive values	3.48 (0.01)	3.48 (0.02)	3.22 (0.02)	3.40 (0.02)	3.45 (0.01)	21.92	4	5.48	28.85^**^	a, b, e > d (= e) > c
Social competences	3.30 (0.02)	3.07 (0.02)	3.33 (0.02)	3.16 (0.02)	3.22 (0.02)	25.51	4	6.38	26.90^**^	a, c > d, e > b
Positive identity	3.41 (0.02)	3.21 (0.03)	2.90 (0.03)	2.97 (0.03)	3.09 (0.02)	118.23	4	29.56	65.85^**^	a > b > e > c, d

*Gender and age were controlled for; M (SE), Mean (standard error); SS, Sum of Squares; MS, Mean Square*.

* *p < 0.05,*

** *p < 0.01.*

a *Ghana,*

b *Kosovo,*

c *Norway,*

d *Slovenia,*

e *Turkey*.

Regarding internal developmental assets, participants from Kosovo and Slovenia had the lowest levels of Commitment to learning (*p* = 1.00), while participants from Norway reported the highest levels of this asset, followed by Ghana (*p* = 0.005) and Turkey (*p* < 0.001). Furthermore, participants from Ghana, Kosovo, and Turkey (*p*s > 0.05) had the highest Positive values among all countries, followed by Slovenia who did not differ from Turkey (*p* = 0.388). Norwegians reported having the lowest Positive values among all included countries. As for Social competencies, participants from Ghana and Norway reported the highest Social competencies among all countries (*p* = 1.00). They were followed by Turkey (*p*s < 0.001), who were followed by participants from Slovenia. Participants from Kosovo had the lowest Social competencies. Lastly, countries were compared in Positive identity. Participants from Ghana were once again the ones with the highest internal asset (all *p*s < 0.001). Participants from Kosovo had a higher Positive identity than participants from Turkey (*p* = 0.009), followed by Norway and Slovenia who did not differ from one another (*p* = 0.708).

To consider the differences in the 5Cs across countries, MANCOVA was employed (Λ = 0.81; *F* = 35.30; *p* < 0.001; partial η^2^ = 0.05). The Bonferroni *post-hoc* tests revealed that countries differed in all of the 5Cs (see Table 6). Regarding Competence, participants from Turkey had the highest levels of Competence among all countries (*p*s < 0.05), followed by participants from Kosovo (*p* = 0.045). They were followed by participants from Slovenia, Portugal, and Ghana. Participants from Ghana did differ from Slovenia (*p* < 0.001) and were no different from Portugal (*p* = 1.00), while participants from Slovenia did not differ from participants from Portugal (*p* = 1.00). Concerning Confidence, participants from Ghana had the highest levels of Confidence in comparison with all included countries (all *p*s < 0.001) and were followed by participants from Kosovo and Turkey who did not differ from one another (*p* = 1.00). Participants from Slovenia had the same levels of Confidence as participants from Portugal (*p* = 1.00). As for Character, participants from Ghana once again reported the highest levels of the PYD outcome in comparison with all included countries (all *p*s < 0.01). They were followed by participants from Turkey (*p* = 0.004) who had higher Character than participants from Kosovo, Portugal, and Slovenia (all *p*s < 0.05). The latter did not differ from one another (*p*s = 1.00). Concerning Caring, participants from Ghana, Kosovo, and Turkey had the highest levels of this PYD outcome and did not differ from one another (all *p*s > 0.05). They were followed by participants from Slovenia and Portugal (*p* = 1.00). Regarding Connection, there were no clear distinctions among countries since participants from Ghana, differed only from participants from Slovenia while participants from Turkey differed from participants from Portugal and Slovenia (*p*s < 0.05) while having higher levels of Connection. Therefore, participants from Portugal and Slovenia had lower levels of Connection.

**Table 6:** 5Cs by Country: MANCOVA.

**Variable**	**Ghana^**a**^*M (SE)***	**Kosovo^**b**^*M (SE)***	**Portugal^**c**^*M (SE)***	**Slovenia^**d**^*M (SE)***	**Turkey^**e**^*M (SE)***	***SS***	***df***	***MS***	***F***	**Group differences**
Competence	3.46 (0.02)	3.62 (0.03)	3.40 (0.04)	3.32 (0.03)	3.74 (0.03)	57.75	4	14.44	32.29^**^	e > b > a = c (= d)
Confidence	4.26 (0.03)	4.02 (0.03)	3.64 (0.05)	3.64 (0.03)	3.98 (0.03)	173.57	4	43.39	83.57^**^	a > b, e > c, d
Character	4.20 (0.02)	3.87 (0.02)	3.90 (0.04)	3.93 (0.02)	4.09 (0.03)	49.63	4	12.41	37.73^**^	a > e > b, c, d
Caring	4.24 (0.03)	4.22 (0.03)	3.98 (0.05)	4.08 (0.03)	4.28 (0.03)	24.27	4	6.07	10.75^**^	a, b, e > c, d
Connection	3.81 (0.02)	3.81 (0.03)	3.72 (0.04)	3.70 (0.03)	3.90 (0.03)	12.65	4	7.28	19.90^**^	a, b, e > c (= a, b), d (= b, d)

*Gender and age were controlled for; M (SE), Mean (standard error); SS, Sum of Squares; MS, Mean Square;*

* *p < 0.05,*

** *p < 0.01.*

a *Ghana,*

b *Kosovo,*

c *Norway,*

d *Slovenia,*

e *Turkey*.

## Discussion

PYD addresses adolescent development by emphasizing strengths rather than deficits and views positive development as an interaction between an active, engaged, and competent person and a receptive, supportive, and nurturing environment (Damon and Gregory, 2003). To thoroughly examine positive youth development worldwide it is crucial to include various countries in the study to focus on the countries' ecology in terms of its political and social context. Therefore, this paper aimed to investigate the distribution of developmental assets and the 5 Cs among youth from six countries (Ghana, Kosovo, Norway, Portugal, Slovenia, and Turkey) with particular focus on their countries' contrasting backgrounds and employing a narrative approach to discuss young people's strengths and opportunities through the lens of the social and political context in which they live.

The analysis revealed a complex pattern regarding the distribution of the developmental assets or the 5 Cs in different countries, suggesting that contextual differences among them may influence youth's development. Developmental assets were examined as a foundation for positive youth development. When considering internal assets, participants from Ghana seemed to have higher levels of internal assets (i.e., positive values, social competencies, positive identity) together with participants from Norway who have the highest commitment to learning. However, participants from Norway were found to report the lowest levels of positive values among all countries. The internal assets in other countries seem to vary as well. External assets such as Support, Empowerment or Boundaries and expectations from family, schools, and society also varied based on the country's economic, social, and political context. Slovenian youth reported higher levels of support compared to their peers. Besides having higher support, youth from Slovenia also felt most empowered. As for boundaries and expectations, differences among countries were not that clear. While youth from Ghana, Slovenia and Turkey, followed by Norway, reported that they used their time most constructively, youth from Kosovo reported not being included in extracurricular activities to this extent.

If developmental assets represent a cornerstone for positive youth development, the 5 Cs can be considered as a consequence of having sufficient resources in youth contexts and personal strengths. Regarding all of the 5 Cs, youth in Ghana reported having the highest confidence and character. Furthermore, youth from Ghana, Kosovo, and Turkey are more caring and connected to others than participants from other countries. Nevertheless, it is crucial to point out that the assets in each country were high, indicating that youth perceive their unique contexts as supportive and engaging. The results for each included country are discussed separately below, with suggestions for culturally-relevant intervention development strategies. While this paper does not intend to make assumptions about the capacity of developmental assets to predict outcomes for young people, we relate the strengths and opportunities existing in each context to their utility in designing relevant interventions. As such, each country section provides details about the pattern of assets within that country and outlines recommendations for designing interventions that draw on available assets in order to more effectively engage young people.

### Ghana

Ghana has one of the most rapidly growing youth populations in the world, constituting about 35% of the country's total population (Tagoe and Oheneba-Sakyi, 2015). With a youth population that is estimated to double by 2030 (Tagoe and Oheneba-Sakyi, 2015), youth are considered a critical human resource and of paramount importance to the national development agenda. In recognition of the importance of investing in young people, the Ghanian government has adopted a youth-focused strategy to boot education and employment, with the establishment of the National Youth Employment Programme (NYEP) for skill training and career development, and the National Entrepreneurship and Innovation Plan (NEIP) to support young Ghanian entrepreneurs (Ile and Boadu, 2018). Indeed, Ghana has recorded a steady decline in youth unemployment rate in recent times (Ampadu-Ameyaw et al., 2020). All these positive youth initiatives appear to be reflected in Ghanaian youth's scores, which was relatively higher than other external assets among this group. Young people also reported high social competencies, a positive sense of personal identity, and higher confidence, which could be due to the significant investments in the empowerment of youth, which featured as a focal point in most government efforts and most notably in the National Youth Policy of 2010 (Ile and Boadu, 2018).

As a collectivist society where child care is a shared responsibility of the closely interknit extended family, young people enjoy strong interpersonal connections (Dzramedo et al., 2018). This is buttressed by the oft-repeated African proverb that “a single hand cannot raise a child” (Dzramedo et al., 2018). With youth development thus firmly embedded within the family system, social responsibility and a sense of loyalty toward others are firmly instilled in young people (Dzramedo et al., 2018). This corresponds to young Ghanian's high scores on positive values, caring for others, and respect for societal norms as observed in this study.

When designing interventions for youth in Ghana, it would be worthwhile to utilize the developmental assets young Ghanian's have; such as strong bonds with their families and communities, their sense of caring for others and their respect for social norms. Some successful interventions have used these strengths through programs that engage youth peer educators to provide sexual health education (Ghana, 2002) and integrate youth interventions within community organizations (Bandy et al., 2008). Considering the emphasis on cultural values, youth interventions could also be framed within the scope of local traditions and by incorporating cultural elements.

### Kosovo

Kosovo as a country in transition faces significant development challenges as one of the poorest countries in Europe in the wake of war and its newfound independence (Smits and Permanyer, 2019). With a strong youth demographic representing 42% of the entire population (Smits and Permanyer, 2019), Kosovo's economic growth is tied to its youth development policies. However, the youth unemployment rate of 61% and limited opportunities in the workplace are major barriers to this objective (Smits and Permanyer, 2019).

Young Kosovans reported low scores on external assets of feeling empowered and supported by their community, as well as a lack of clear boundaries and positive role models. These findings can be explained by prior research that describes young people's low level of trust in government institutions and disappointment with the poor implementation of the Kosovo Youth Action Plan (Senyuva, 2017). Young people have also described failures with the education system due to the lack of infrastructure and resources, inadequate teaching methods, and lack of extracurricular activities (Islami, 2018). Despite these drawbacks, young people in the study scored high on internal assets of caring and feeling empathy for others. Kosovan youth also reported high levels of positive identity and self-esteem which generally promote positive development. However, in light of the evidence of associations between high self-esteem among Kosovan youth and negative outcomes such as anxiety, depression, and substance use (Fanaj and Melonashi, 2014), this asset should be treated with consideration (Radović and Jaredić, 2014).

As a growing economy and newly developing country, Kosovo's capacity to fund and implement nationwide youth development programs is limited. Similarly, resource-limitations in the education sector reduce its potential to host scalable school-based interventions. Instead, researchers and policy-makers may consider programs that engage existing resources by training parents to facilitate asset-building and thus, promote the development of young people. Considering the evidence on the negative outcomes related to self-esteem, further research is required to understand these pathways among young people in Kosovo.

### Norway

Norway has a relatively small population with young people making up close to 20% of the total strength (Norwegian Institute of Public Health, 2018). While Norway doesn't have a youth law, its comprehensive youth policy is implemented efficiently in a top-down approach (Norwegian Institute of Public Health, 2018). Young Norwegians generally enjoy a higher life satisfaction than other Nordic countries, with lower social inequalities (Due et al., 2019). However, high-income countries, such as Norway, face a higher risk for mortality and disability due to mental health and substance use disorders (Due et al., 2019).

Norway, a highly developed country, has no shortage of resources to ensure the optimal development and well-being of its youth. With significant funding allocated to investing in youth development and a plethora of youth-focused interventions successfully launched around the nation (Norwegian Institute of Public Health, 2018), it is not surprising that young people scored highly on the external asset of “empowerment.” Norwegian youth also scored higher than their peers on the internal asset of social competencies and commitment to learning.

Youth-focused programs in Norway often take advantage of the strong government support and funding, by integrating interventions in public health services or public schools. This approach has proved to be effective in tackling issues such as mental health (Bjørnsen et al., 2018) and substance use (Jøsendal et al., 1998; Strøm et al., 2015). Considering the cultural ubiquity of technology and a lower degree of interdependence, self-guided digital interventions could be considered for Norwegian youth.

### Portugal

With a declining youth population that makes up 10% of the total population, Portugal faces an estimated drop in population to below 10 million in 2031 (Moreira and Filipa, 2016). Portuguese dwindling youth population faces challenges in breaking into an already saturated workplace; ~18% of young people (15–24 years old) are neither in employment nor in education or training (NEETs) (Maynou et al., 2020). Mental health among Portuguese youth has also shown some alarming trends in the last decade with 20% of adolescents and young adults presenting psychiatric disorders (Marques and Brissos, 2014) and suicide featuring as one of the leading causes of death among Portuguese youth (Marques and Brissos, 2014). However, the Portuguese government has taken swift steps to address these concerns with the implementation of a National Mental Health Plan that puts youth mental health promotion at the forefront of its agenda (de Almeida, 2009).

An evaluation of Portuguese youth's reporting on the 5 Cs indicated a high level of character and caring. This is in accordance with recent policy trends in Portugal that have focussed on empowering young people to take an active role in the national health promotion efforts and provide peer-to-peer support (De Matos et al., 2018). A notable example is the nation-wide implementation of the Dream Teens project that successfully encouraged young people's participation and active citizenship through the positive youth development framework (Frasquilho et al., 2018). The findings of this study also indicated that young Portuguese have greater respect for social norms. This could be explained by their increased civic engagement garnering an improved awareness of social responsibility.

Current youth-focused programs have successfully operationalized young people's strengths to develop youth-led interventions (Carvalho, 2017) embedded within Portugal's youth culture, such as the innovative SURF.ART program which uses a surfing-intervention to catalyze youth development (Gomes et al., 2020). Further intervention programs could be integrated within youth activities to build internal assets of caring and a strong positive identity, while family-targeted interventions could strengthen their support systems and increase young people's reservoir of external assets.

### Slovenia

Young people in Slovenia represent a gradually declining proportion of the population, with youth aged 15–25 years accounting for just 10% of the general population in 2017 (Bučar et al., 2018). This decline has been accompanied by growing unemployment rates and a heightened risk of poverty among young people (Senekovič, 2016). Mental health statistics of Slovenian youth reveal disturbing trends of high suicide rates, with suicide ranking as one of the three main causes of death among young people in the country (Flere et al., 2014).

In the context of job insecurities and growing unemployment, it is unsurprising that young people in our study experienced lower feelings of self-competencies, a shaky sense of their self-worth, and most concerningly a lower motivation for academic and work commitments. However, substantial funding from the EU and coordinated efforts by various government ministries to encourage young people to active citizenship were reflected in our findings which indicated that Slovenian youth reported feeling confident and highly empowered by their society. Slovenia also boasts close family relationships and community ties and provides young people with considerable family support. Parental relationships have a demonstrated influence on positive development, with family influences predicting better employment outcomes and closer parental connections protecting against anxiety (Kozina et al., 2020). Our study found that Slovenian youth scored high in domains of external support, such as empowerment and support.

This suggests that family or community-based interventions which build on existing interpersonal networks could be particularly beneficial among Slovenian youth. In light of the strong government investment in youth, interventions could be structured within the framework of public services. This will allow implementers to more effectively design and deliver interventions using the existing government-funded infrastructure.

### Turkey

Turkey has a dynamic, young population, with youth between 15 and 29 years making up a solid 24.4% of the Turkish population (Bakar et al., 2017). However, with a declining youth demographic and high unemployment rates among young people (Bakar et al., 2017), there is a need to scale up efforts for youth development. Of the 12.9 million young people, 20.8% are unemployed despite improvements in the education sector (Bakar et al., 2017). Gender disparities are also apparent with 6% of young women being illiterate and representing lower workplace participation (Susanli, 2017) while increasing privatization of education causes divisions based on socio-economic groups (Duman, 2008).

Turkish youth participating in this study reported high degrees of positive family bonds and caring for others, which is unsurprising in the context of family-oriented social norms (Kagitcibasi, 2007). Additionally, high unemployment and lower financial independence lead young people to live with families and rely on their support for a longer period (Özdemir et al., 2013). As a conservative society, Turkish families also tend to be highly involved in the personal lives of young people (Özdemir et al., 2013). Despite this, Turkish youth in this study scored low on the “boundaries and expectations” asset, which describes young people having clear roles, positive influences, and encouragement. However, youth also possessed strong integrity, respect for social norms, and had a positive view of their competencies, which could be associated with the support and guidance provided by family systems (Kagitcibasi, 2007). Young people also reported lower opportunities to use their time constructively, which could be a result of lower access to quality education and opportunities to engage in extracurricular activities.

### Limitations and Suggestions for the Future Research

This study has several strengths as it includes various countries from Europe, Africa, and Asia with different political and social contexts, however, there are some limitations worth mentioning. There was a variation in subsample size since only 246 students from Portugal were included in the study and were compared to larger subsamples from other countries. Furthermore, included subsamples are not nationally representative as in the majority of countries convenient samples were collected. Moreover, internal consistencies for Constructive use of time were unacceptable in every included country, suggesting this has to be taken into account when interpreting the results. Additional differences in scales might have occurred during the translation process in each country since the original scale was in English and was adapted to the US context. Moreover, SES, parents' educational level and religion should have been included as control variables as well since between countries and within countries differences are immense. In light of the above-mentioned limitations, more rigorous procedures that ensure similarities of study scales and larger subsamples are recommended in future research.

While the two PYD models studied in this chapter- the Developmental Assets and the 5 Cs model, were independently developed and tested, they appear to have conceptual similarities. For example, the external developmental asset of “support” described as young people experiencing “support, care, and love from their families, neighbors, and many others,” bears similarities to “connection” from the 5 Cs model which is defined as “positive bonds with people and institutions.” Similarly, the internal asset “positive identity” characterized by “belief in one's self-worth and feeling control over the things that happen,” has similar language as “confidence” which is described as an “internal sense of overall positive self-worth and self-efficacy.” Despite such theoretical links between these two prominent frameworks, there is no empirical investigation of the associations between these constructs. Furthermore, this study observed interesting discrepancies between these constructs in a few instances. For example, despite Slovenian youth reporting high levels of support, they also experienced lower connections in comparison with other countries. While an investigation of the conceptual similarities between these constructs was beyond the scope of this work due to the inconsistencies in our sample (only participants from Ghana, Kosovo, and Slovenia completed both questionnaires), further research is recommended to explain their relationship and such discrepancies.

## Conclusion

This paper studies developmental assets and the 5 Cs among youth from six countries; Ghana, Kosovo, Norway, Portugal, Slovenia, and Turkey, which represent the Global North and Global South, different cultural influences, and vastly different economic and political situations. These contextual factors play an important role in influencing young people's development. This study reported that countries like Norway and Slovenia have a strong ability to empower young people, while in countries like Ghana, Kosovo, and Slovenia, young people enjoy support from family systems and other social relationships. These assets should be operationalized as suitable platforms for delivering more feasible and sustainable interventions. In contexts with strong youth policies and existing youth development programs, initiatives that are integrated within primary care, education systems or existing intervention have the potential to reach and influence young people. On the other hand, interventions embedded in the fabric of the family or broader community have the potential to be vastly impactful in contexts with higher social support systems.

Similarly, young people's internal assets could also be used to guide intervention development. Youth with strengths like caring for others, confidence, and social competencies, as seen in Norway and Ghana, could be encouraged to lead youth-focused initiatives through peer-delivered interventions. Developing contextually relevant interventions embedded in the local culture can be hugely beneficial in effectively engaging young people and promoting positive developmental outcomes.

## Data Availability Statement

The data analyzed in this study is subject to the following licenses/restrictions: The datasets used in this study are part of the cross-national PYD project and can be requested from the concerned author. Requests to access these datasets should be directed to nora.wiium@uib.no.

## Ethics Statement

The studies involving human participants were reviewed and approved by Norwegian University Ethical Committee Ethical Board of Ozyegin University Ethics Commission of the Medicine Academic Center of Lisbon Ethics Committee for Humanities at the University of Ghana and Ethics committee of Faculty of Arts, University of Maribor. Written informed consent to participate in this study was provided by the participants' legal guardian/next of kin.

## Author Contributions

All authors listed have made a substantial, direct and intellectual contribution to the work, and approved it for publication.

## Conflict of Interest

The authors declare that the research was conducted in the absence of any commercial or financial relationships that could be construed as a potential conflict of interest. The handling editor is currently organizing a Research Topic with one of the authors NW.

## Publisher's Note

All claims expressed in this article are solely those of the authors and do not necessarily represent those of their affiliated organizations, or those of the publisher, the editors and the reviewers. Any product that may be evaluated in this article, or claim that may be made by its manufacturer, is not guaranteed or endorsed by the publisher.
